# Fungal Endophyte Diversity in *Sarracenia*

**DOI:** 10.1371/journal.pone.0032980

**Published:** 2012-03-13

**Authors:** Anthony Glenn, Michael S. Bodri

**Affiliations:** 1 Toxicology and Mycotoxin Research Unit, Russell Research Center, United State Department of Agriculture Agricultural Research Service, Athens, Georgia, United States of America; 2 Department of Biology, North Georgia College & State University, Dahlonega, Georgia, United States of America; Centro de Investigación y de Estudios Avanzados, Mexico

## Abstract

Fungal endophytes were isolated from 4 species of the carnivorous pitcher plant genus *Sarracenia*: *S. minor*, *S. oreophila*, *S. purpurea*, and *S. psittacina*. Twelve taxa of fungi, 8 within the Ascomycota and 4 within the Basidiomycota, were identified based on PCR amplification and sequencing of the internal transcribed spacer sequences of nuclear ribosomal DNA (ITS rDNA) with taxonomic identity assigned using the NCBI nucleotide megablast search tool. Endophytes are known to produce a large number of metabolites, some of which may contribute to the protection and survival of the host. We speculate that endophyte-infected *Sarracenia* may benefit from their fungal associates by their influence on nutrient availability from within pitchers and, possibly, by directly influencing the biota within pitchers.

## Introduction

**Figure 1:**
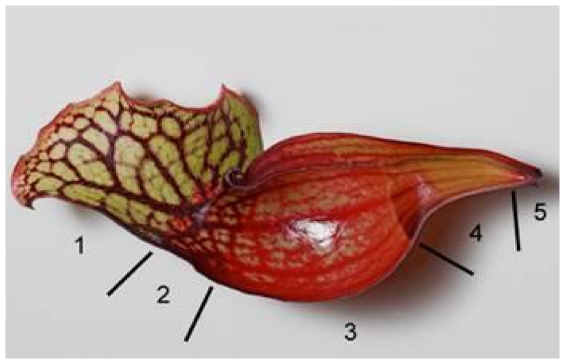
Sagittal section of *Sarracenia purpurea* illustrating the 5 Hooker zones. Zone 1, attraction; zone 2, conduction; zone 3, glandular; zone 4, digestion/absorption; zone 5, no known function although it has some similarity to zone 3. (Photograph © Barry Rice, Sarracenia.com).

The carnivorous North American genus *Sarracenia* is represented by 9 species of wet savannah and bog-associated herbaceous perennial plants characterized by hollow cylindrical or tubular pitfall-trap leaves bounded by a lid or hood and arranged in circular rosettes. The modified leaves (pitchers), typically 1/6 to 1/3 fluid-filled, are attractive to arthropods due to their conspicuous color and secreted nectar. Pitchers are differentiated into 4–5 Hooker zones [1], as described in [2], each with a specialized function (Fig. 1). Zone 1, attraction, is the pitcher lid, containing nectar glands. Zone 2, conduction, includes the pitcher lip and extends partially into the tubular portion of the pitcher. It produces nectar as well as a powdery ablative waxy material that can interfere with arthropod locomotion. Zone 3, glandular, contains sunken glands that produce digestive enzymes. Zone 4, digestion/absorption, is submerged by the fluid contained within the pitcher. The lack of cuticle within this zone enhances nutrient absorption. Zone 5, present only in *S. purpurea* L. (Purple Pitcher Plant) and *S. rosea* Naczi, Case and R.B. Case (Burke's Southern Pitcher Plant) and at the bottom of the trap, has no known function although it has some similarity to zone 3. Although prey capture differs slightly among the species, the ultimate fate of the prey is to die within the pitcher, generally at the bottom, where they are digested by enzymes secreted by the plant [3] and/or by the resident microbial and small invertebrate community [4]. Zone 4 has received the majority of scientific attention because, in addition to the digestive enzymes produced by the plants, it contains “a microcosm composed of larval insects, fungi, algae, rotifers, nematodes, and bacteria that, together, ultimately break down nutrients from insect prey for the plant” [5]. Additionally, this microcosm is potentially amenable to analysis and manipulation of its inhabitants, allowing for detailed experimental study of their role and impact.

The evolution of carnivory is partially attributed to the adaptation and persistence of these plants to nutrient-poor habitats [6]. Beginning with Darwin [7], multiple studies have addressed the different facets of carnivory and its independent evolution 6 times in 5 angiosperm orders [8]. Givnish et al. [9] and Benzing [10] postulated, in cost-benefit models, that carnivory evolved to provide increased nutrients and impart an energetic advantage to plants found in environments limited in nutrients but not water and sunlight. In *Sarracenia*, these studies typically examine the composition and role played in nutrient availability, transformation and allocation by the microecosytem that exists within pitchers [4], [5], [11]–[15].

Little attention has been paid to nutrient capture by roots of carnivores. Most species are restricted to extremely nitrogen-limited habitats and subsequently, in the case of *Sarracenia*, acquire up to 80% of their nitrogen from prey captured in their leaves rather than from weakly developed root systems [15]. Because construction costs of traps is comparable to that of rhizomes and roots in *Sarracenia* [16], it is advantageous for these plants to invest in structures that have a better chance of nutrient capture. The poorly developed root structures and primary mode of nutrient acquisition via the modified leaves led to the belief that carnivorous plants lacked mycorrhizal associations even though the roots may occasionally be colonized by facultative mycorrhizas [17]. Fungal hyphae that colonize plant root tissue without any deleterious effects are often endophytic species [18].

Rodriguez et al. [19] are of the opinion that all plants in natural ecosystems are symbiotic with fungal endophytes that, in turn, influence their evolution and ecology [20], the community structure of the plants [21], and the other organisms associated with the plant [22]. Bioactive compounds [23] and enzymes [24] produced by endophytes are thought to possibly influence nutrition and/or growth of the endophyte. These same chemicals could theoretically affect the microcosm present within the pitchers of *Sarracenia* and therefore nutrient availability to the plant. Because endophytes have the ability to influence the communities associated with an infected plant, this study was undertaken to ascertain the presence of *Sarracenia* pitcher endophytes and the restricted distribution, if any, of the endophytes to the different Hooker zones. In this preliminary study we wish to hereby report the existence of fungal endophytes within the leaf tissue of multiple *Sarracenia* species and further report the first instance of carnivorous plant endophytes cultured specifically from leaves. In addition, we speculate on the possible roles these endophytes may play in regards to the digestive processes and nutrient utilization of their host plants.

## Results

Of the 7 individual pitchers collected from *S. oreophila*, bacteria were cultured on brain-heart infusion from 4 individual plants and only from Hooker zone 3–4 after 4 days of incubation at 25°C. As only 10 morphologically similar bacterial colonies were produced from over 168 leaf sections of *S. oreophila*, no further attempts were made to isolate bacteria from other species of pitcher plant. All subsequent investigations targeted growth and isolation of fungal endophytes.

**Figure 2:**
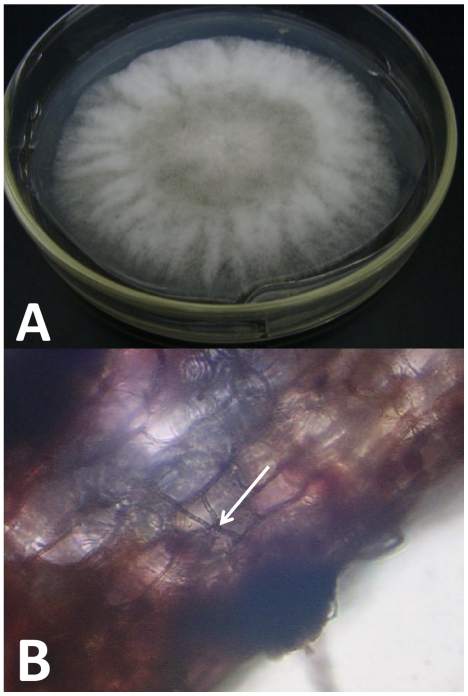
Gross and histological appearance of a fungal isolate of *Sarracenia oreophila*. A, Appearance of *Colletotrichum gloeosporioides* #3 isolated from *S. oreophila* on potato dextrose agar and B, in a hand-cut thick tissue section (400× magnification) stained in 1% acid fuchsin. Note the fungal hyphae growing between cells denoted by the arrow.

Fungi were isolated from all 4 species of *Sarracenia* and from Hooker zones 2–3 and 3–4, with almost 100% of pieces positive for endophytes (data not shown). Histological sections were examined microscopically for the presence of fungal hyphae. Vegetative fungal hyphae were observed and appeared to be confined to the intercellular spaces (Fig. 2).

*S. purpurea* harbored the greatest diversity of endophytes based upon the DNA sequencing of dissimilar morphotypes as no isolate was represented more than once and none were isolated from the other *Sarracenia* species (Table 1). In contrast, *S. minor* was infected with only a single species, *Colletotrichum gloeosporioides* (Penz.) Penz. and Sacc. Overall, *Colletotrichum* spp. was the most common genus, infecting at least one plant of each *Sarracenia* species. Three ITS alleles were identified among the isolates of *C. gloeosporioides*. Two alleles were identified in the isolates from *S. minor* and differed at 2 nucleotide positions. These 2 alleles each differed at 11 nucleotide positions compared to the third *C. gloeosporioides* allele in the 2 isolates from *S. oreophila*.

**Table 1:** Fungal endophyte isolates from *Sarracenia* identified based on sequence data from the internal transcribed spacer regions of nuclear ribosomal DNA (ITS rDNA).

*Sarracenia* species and individual plants sampled	Endophyte strain #	Endophyte identification (GenBank accession with greatest identity)^a^	GenBank accessions of fungi from this study
*S. minor*			
#2	SM2-1	*Colletotrichum gloeosporioides* #1	JF288537
		(EF423526)^b^	
#3	SM3-1	*Colletotrichum gloeosporioides* #1	JF288538
		(EF423526)^b^	
#5	SM5-1	*Colletotrichum gloeosporioides* #2	JF288539
		(EF423526)^b^	
#7	SM7-1	*Colletotrichum gloeosporioides* #2	JF288540
		(EF423526)^b^	
*S. oreophila*			
#1	SO1-1	*Colletotrichum gloeosporioides* #3	JF288541
		(AJ301907)^b^	
#2	SO2-1	Xylariales sp.	JF288542
		(GQ906959)	
#5	SO5-1	*Colletotrichum gloeosporioides* #3	JF288543
		(AJ301907)^b^	
#6	SO6-1	Pleosporales sp.	JF288544
		(AF525674)	
*S. psittacina*			
#2	SPs2-1	Basidiomycete sp. #1	JF288546
		(EF694649)	
#5	SPs5-1	Basidiomycete sp. #2	JF288547
		(EU622841)	
#7	SPs7-1	*Penicillium* sp.	JF288548
		(HM043803)	
#8	SPs8-1	*Colletotrichum gloeosporioides* #1	JF288549
		(EF423526)^b^	
*S. purpurea*			
#1	SPu1-1	*Paraconiothyrium/Coniothyrium* sp.	JF288550
		(EU821483)	
#2	SPu2-1	*Colletotrichum acutatum*	JF288551
		(AJ301905)	
#4	SPu4-1	*Phomopsis* sp.	JF288552
		(EF432292)	
#6	SPu6-1	Basidiomycete sp. #3	JF288553
		(AJ279465)	
#7	SPu7-1	Basidiomycete sp. #4	JF288554
		(AB566279)	
#8	SPu8-1	*Cryptosporiopsis actinidiae*	JF288555
		(AY359233)	

a GenBank accessions having the greatest identity (>97%) to the *Sarracenia* endophytes based on megablast of ITS rDNA sequences. Consideration was given only to accessions with voucher strains. All megablast ITS rDNA queries identified GenBank accessions with E-values of zero, except for endophyte strain SPs5-1, which was most similar to basidiomycete accession EU622841 but with an E-value of 1e-176.

b Three different *C. gloeosporioides* alleles were identified. Alleles #1 and #2 differed only at two nucleotide positions, while they both differed from allele #3 at 11 nucleotide positions.

**Figure 3:**
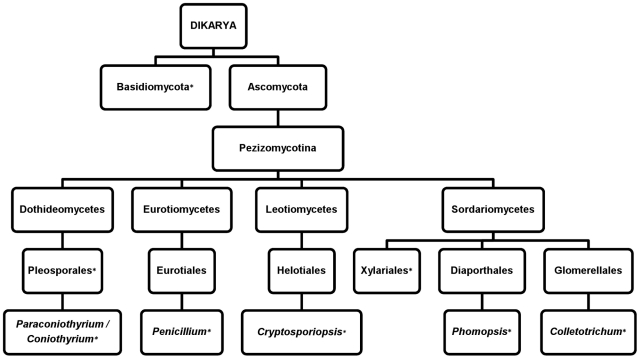
Representational phylogeny showing the taxonomic distribution of fungal endophytes isolated from *Sarracenia*. Fungi isolated in this study are denoted by an asterisk [*] following the taxa name.

Both the Ascomycota and Basidiomycota phyla were represented by endophytes from *Sarracenia* leaves. Four unidentified basidiomycetes were isolated from *S. psittacina* and *S. purpurea* (2 species each). All megablast ITS rDNA queries identified GenBank accessions with E-values of zero (identities >97%), except for endophyte strain SPs5-1 (Table 1), which was most similar to a basidiomycete accession (EU622841) with an E-value of 1e-176, which indicates this *Sarracenia* endophyte may not have been previously sequenced and deposited in the GenBank database or may represent a novel taxon. In addition to the ascomycete *Colletotrichum* of the class Sordariomycetes (order Glomerellales), 5 additional orders from 4 classes were represented: Pleosporales (Dothideomycetes); Diaporthales and Xylariales (Sordariomycetes); Eurotiales (Eurotiomycetes); and Helotiales (Leotiomycetes) (Fig. 3).

## Discussion

The highly diverse nonclavicipitaceous endophytes can be divided into 3 functional groups based upon host colonization patterns, mechanism of transmission, biodiversity levels within plant tissues and ecological function [19]. All classes have broad host ranges but class 3 exist as highly localized independent infections restricted to above ground plant tissues allowing for extremely high in planta biodiversity while class 4 are primarily ascomycetous fungi restricted to roots where they form inter- and intracellular hyphae and microsclerotia and are capable of extensive tissue colonization [19]. Members of class 2, species within the Ascomycota or Basidiomycota such as the fungi we have isolated from leaves of *Sarracenia* species, infect and colonize via appressoria or by direct penetration by hyphae [25]. Some are known to confer fitness benefits to their host, especially if there are habitat-specific selective pressures placed on the host [26]. These selective pressures have been associated with pH, temperature, drought, and salinity but also likely include growth in nutrient-deficient soils. Class 2 endophytes have also been demonstrated to increase plant biomass under stressful conditions [19]. Colonization of *Sarracenia* plants by endophytes in the spring may account for the subsequent increase in pitcher size observed over the course of the growing season particularly for *S. leucophylla*. Many endophytes produce phytohormones such as indole-3-acetic acid, cytokines, and other plant growth-promoting substances [27]. We also speculate that endophyte-infected plants benefit from their fungal associates by their influence on nutrient availability from within pitchers and, possibly, by directly influencing the biota within the pitchers. Tan and Zou [27] postulate that horizontal gene transfer may explain why some endophytes are capable of producing phytochemicals characteristic of the host. Although speculative at this point, carnivorous host plants may have co-evolved along with fungi and now rely on endophytes for production or augmentation of levels of some of the digestive enzymes found within their pitchers.

Cost-benefit models predict that carnivory could result in an increased rate of photosynthesis manifested in one form as an increase in total leaf mass, but experiments to support this have been equivocal [13], [28], [29]. While most nutrient supplementation studies do identify a significant increase in growth, excess nutrients may not lead directly to increased photosynthetic rates [8]. *Cyperus erythrorhizos* Muhl. (Redroot Flatsedge), a C4 plant growing in nutrient deficient wetlands, habitat similar to that for many *Sarracenia*, have a high photosynthetic nitrogen use efficiency (PNUE) [30] in contrast to the low PNUE of *Sarracenia* [13], which are likely C3 plants. The activity of endophytes within pitcher plant leaf tissue may explain the PNUE observations in comparison to non-carnivorous plants growing in similar habitats. The presence of endophytes could also help discern why discordant data exists between the construction costs of carnivorous leaves versus phyllodia, help elucidate how carbon derived from prey is utilized, and further clarify the relationship between plant biomass and photosynthesis in nutrient-manipulated plants. For example, non-feeding *Sarracenia* are phosphorus-limited or nitrogen+phosphorus co-limited while artificially fed plants are more strongly nitrogen-limited [13]. Of the nitrogen available from prey, 60% is sequestered by bacteria in the pitchers [4], [31]. Over a growing season, plants will rely on prey-based phosphorus to boost photosynthetic efficiency due to nitrogen-limitation from bacterial sequestration [13]. It has been observed that endophyte growth limitation by lack of an adequate nitrogen source or phosphate components of the nutritional environment leads to the synthesis of secondary metabolites [32] and these metabolites, released into the plant tissue or pitcher milieu, can alter growth characteristics if they are plant growth regulators or impact the micro-organisms present within the pitcher if antimicrobial in nature.

The endophyte isolates of *Sarracenia* may contribute to their hosts' fitness by means of the production of biologically active compounds. Basidiomycetous endophytes are rarely isolated from higher plants and these are often orchid mycorrhizas [33]. While many of these latter species are associated with white rot and brown rot of trees and may be saprobes or latent pathogens, their production of lignocellulolytic enzymes and potential to produce bioactive secondary metabolites may favor their association with *Sarracenia* pitchers. *S. purpurea*, for example, from which two basidiomycete isolates were obtained, has open pitchers that often accumulate plant debris. Degradation of captured plant debris by these fungi would be beneficial to host and endophyte.

Every Ascomycota endophyte taxa isolated from *Sarracenia* in this study has been proven to produce biologically active compounds. Two isolates representative of the Pleosporales were found, one being a member of the polyphyletic anamorph *Paraconiothyrium/Coniothyrium* and the other being an unidentified taxon. Members of the Pleosporales exhibit a diversity of habits, including parasites, saprobes, and endophytes [34]. Preussomerins, isolated from various fungi, including *Edenia gomezpompae* M.C. González, Anaya, Glenn, Saucedo, and Hanlin, a Pleosporales endophyte of *Callicarpa*, possess a wide range of biological properties, including antibacterial, algicidal, herbicidal, antiplasmodial, antitumor, and antifungal activities, including inhibition of other endophytes and phytopathogens [35]. Interestingly, the genus *Paraconiothyrium* was erected to segregate mycoparasitic species from *Coniothyrium* species [36]. An endophyte that utilized other fungi as a food source could influence competition for resources within the flooded and enzyme-poor pitchers of *S. purpurea*, from which this isolate was made.

Endophytic penicillia are widespread and heterogenous, and various species have been reported as endophytes of plants with 20 species of *Penicillium* isolated from the roots of *Picea mariana* (Miller) Britton Sterns, and Poggenburg (Black Spruce) alone [37]. By the end of 1984, over 380 biologically active metabolites were known from *Penicillium* [32] and even more are now known. Of the 6450 biologically active compounds identified from microfungi up through 2009, over 30% have been obtained from *Aspergillus* and *Penicillium* [38] and these compounds exhibit a vast diversity of activity.

*Cryptosporiopsis* spp. (teleomorph *Pezicula* spp.) have had biologically active compounds isolated that exhibit antibacterial, antifungal and algicidal activities [39]. Echinocandin isolated from *Cryptosporiopsis* sp. and *Pezicula* sp. was shown, in vitro, to inhibit pathogens of the respective host plants [40]. Many strains of *Pezicula* synthesize, in vitro, fungicidal compounds such as mullein, mycorrhizin, epi-ethiosolide, cryptosporopsin, and cryptocandin [39], [41] postulated to help it to maintain a mutualistic role with its host.

Potent antifungal agents, the sordarins, are synthesized by members of the Xylariales, a trait more frequently attributed to this group than to any other fungal order [42]. Species of *Xylaria* have been shown to produce potent antifungal and antibacterial metabolites [43]. The Xylariales may either be colonizers that will later decompose cellulose when the host plant dies or be true endophytes, although obvious benefits to the host plant have yet to be documented [44].

*Phomopsis* and *Colletotrichum* are common isolates from dicot leaves and frequently dominate endophyte assemblages of the host [45]. *Phomopsis* spp. are not host specific and exhibit high host variability. The genus is a very rich source of secondary biological compounds with antifungal, herbicidal, algicidal, antimicrobial, and plant growth regulating activities [46].

Fungi of the genus *Colletotrichum* are well documented as significant plant endophytes [47]–[50]. *C. gloeosporioides* in particular has been reported from at least 470 host genera and more than 2000 taxa [51] primarily as a host-specific pathogen although *C. gloeosporioides* is now recognized as a broadly defined species complex that contains multiple clades, each of which may represent a genetically isolated species [52]. Lu et al. [50] isolated metabolites of a *Colletotrichum* sp. from *Artemisia annua* and Zou et al. [23] similarly isolated metabolites of *C. gloeosporioides* from *Artemisia mongolica* that have antimicrobial and antifungal properties. Tan and Zou [27] found that culture broth from *C. gloeosporioides* could promote the growth of host callus. As this genus was represented in every species of *Sarracenia* studied, and isolated multiple times, there is great potential for a mutualistic relationship between this endophyte and host that could be further elucidated by an examination of the secondary products, if any are produced by the fungus and compared to the native metabolites and enzymes produced by sterile plants. Furthermore, some light could be shed on the problem of nutrient limitation and stoichiometry of carnivorous plants should sterile and endophyte-infected plants be utilized and compared under experimental conditions.

It is well established that some of the large number of metabolites produced by endophytes offer a significant benefit to their host plant [27]. These benefits could contribute to the protection and survival of the host by acting as growth regulators, antimicrobials, and mediators of environmental stress. In the case of *Sarracenia*, these metabolites could have an even broader impact due to the unique structure of the trap leaf, which would function to capture and store excreted materials within the pitcher where the chemicals would interact with prey as well as a complex microcosm of associated organisms.

This is the first instance of *Coniothyrium*/*Paraconiothyrium*, *Penicillium*, *Cryptosporiopsis*, *Phomopsis* and *Colletotrichum* spp. ascertained to be endophytes of the family Sarraceniaceae and, to our knowledge, the first report of fungal endophytes of leaves of any carnivorous and proto-carnivorous plant family. The isolation of *Colletotrichum* spp. from multiple *Sarracenia* individuals of all 4 species at locations 300+ miles apart, as well as during different years, strongly suggests that at least this fungal genus is a true pitcher plant endophyte. This study only concerned the isolation of fungal endophytes. Our preliminary assay also indicates a potential diversity of bacterial endophytes as well. The role, if any, of the fungal endophytes we isolated from *Sarracenia* is unknown as is whether they produce any biological compounds that may be of benefit to the plants. At this time it is unknown if these endophytes truly contribute to carnivory in *Sarracenia* and further investigation concerning the role of endophytes and/or their metabolites in successful carnivory is highly desired.

## Materials and Methods

A single mature pitcher was collected from 7–8 different individuals of 4 species of *Sarracenia* (*S. minor* Walter [Hooded Pitcher Plant], *S. oreophila* [Kearney] Wherry [Green Pitcher Plant], *S. psittacina* Michaux [Parrot Pitcher Plant], *S. purpurea*). Sampling of *S. oreophila*, a critically endangered species only found in small isolated populations scattered in 7 non-coastal plain counties in 3 southeastern states, was permitted by the Georgia Chapter of the Nature Conservancy in August 2006. *S. minor* and *S. psittacina* were sampled in July 2009 under permit by the U.S. Fish and Wildlife Service, Okefenokee National Wildlife Refuge. *S. minor* and *S. psittacina* are coastal plain inhabitants. Ranging from extreme southeastern North Carolina to northern Florida, *S. minor* is found in 119 counties in 4 states. *S. psittacina* is irregularly distributed in 67 counties in 6 states. Highlands Biological Station, an interinstitutional research center of the University of North Carolina, permitted sampling of *S. purpurea*, also in July of 2009. *S. purpurea* has the most expansive range of all *Sarracenia*. It is found in 25 states as well as Canada. The individuals sampled in the mountains of western North Carolina are considered a disjunct population of *S. purpurea* ssp. *purpurea*. Populations of all plants sampled were natural stands with minimal human impact. In addition to the temporal isolation by collection date, *S. minor* and *S. psittacina* are geographically isolated from *S. oreophila* and *S. purpurea* by more than 300 miles. *S. oreophila* and *S. purpurea* sites are approximately 50 miles apart and separated by mountains. Species were selected to represent erect (*S. oreophila*, *S. minor*) or decumbent (*S. psittacina*, *S. purpurea*) pitcher habit as well as lids that are open (*S. oreophila*, *S. purpurea*) or overhanging (*S. minor*, *S. psittacina*). These 4 species, in addition to the contrasting morphologies, also differ by means of their digestive physiologies. All pitchers were live (no sign of senescence), firm, and green and assumed to be free of disease. Pitchers were processed for endophyte culture within 24 hours of collection.

A culture dependent approach was utilized to isolate endophytes. This approach, by the nature of the isolation technique, does influence the species of endophyte that can be isolated. Pitchers were cut longitudinally and washed carefully for 1 hr under running tap water with the addition of dishwashing soap. Pitcher halves were then further sectioned to represent Hooker zones 2–3 and 3–4, conduction-glandular and glandular-digestion/absorption respectively. The top and bottommost sections were discarded. These retained sections were surface sterilized by dipping into 95% EtOH for 1 min followed by immersion in a 10% household bleach plus Tween-20 (3 drops/100 ml) solution for 10 min. A second alcohol dip of 30 sec duration preceded a final wash in sterile ultra-pure water. Sterilized leaf sections were cleaved aseptically into a minimum of 12 small segments, approximately 3 mm×3 mm, and placed onto 2% water agar. Sterilized sections of leaf from *S. oreophila* were also placed onto 3.7% brain-heart-Infusion agar (Bacto™ Brain Heart Infusion, Becton, Dickinson and Company, Sparks, MD). Incubation of samples was performed in the dark at 25°C until the outgrowth of endophytes from the cut edges of the leaf material was discerned. Hyphal tips originating from leaf segments on water agar were transferred to Petri dishes containing potato dextrose agar (PDA; Difco™ Potato Dextrose Agar, Becton, Dickinson and Company, Sparks, MD), grown for 8–10 d, and periodically checked for culture purity. Pure cultures were obtained by hyphal tipping onto PDA so that morphology of the fungal culture could be observed. Fungi were grouped into morphotypes based upon appearance on PDA. Selected isolates within dissimilar morphotypes from each *Sarracenia* species were utilized for sequencing.

Representative leaf sections from which fungi were cultured were preserved and stained in 1% acid fuchsin and cleared in 85% lactic acid in preparation for later histological processing. Histology was performed by embedding plant sections in JB-4 (JB-4 Mini Kit, Polysciences, Inc., Warrington, PA) following dehydration in ascending grades of EtOH baths and infiltration of the polymer according to the manufacturer's directions. Sectioning was done with a microtome (Energy Beam Sciences, Inc., JB-4, Energy Beam Sciences, East Granby, CT) utilizing a glass knife or preserved sections were cut by hand with razor blades.

The identity of fungal isolates was determined based on PCR amplification and sequencing of the internal transcribed spacer sequences of nuclear ribosomal DNA (ITS rDNA). DNA was isolated from fungal cultures using a Bio-Rad Aqua Pure Genomic DNA Kit (Bio-Rad Laboratories, Hercules, CA). The ITS rDNA region (ITS1-5.8S rDNA-ITS2) was amplified and sequenced using primers ITS5 and ITS4 as previously described [53]. Sequencing was performed by the United States Department of Agriculture–Agricultural Research Service South Atlantic Area Sequencing Facility (Athens, GA, USA). The DNA sequences were deposited in GenBank (Table 1). Taxonomic identity of strains was assigned using the NCBI nucleotide megablast search tool (http://blast.ncbi.nlm.nih.gov/Blast.cgi). A threshold of 97%, the lowest identity observed for any of the blast searches, was used to determine the identity of the sequences. Only ITS nucleotide accessions associated with voucher strains were considered for taxonomic assignment of the *Sarracenia* endophytes.
